# Supplementary material for: Compatibility between object size and response side in grasping: the left hand prefers smaller objects, the right hand prefers larger objects
Source: PeerJ. 2018 Dec 3;6:e6026. doi: 10.7717/peerj.6026 (PMC6282946; doi:10.7717/peerj.6026)
Supplement: Table S1 — Descriptive statistics of MT and several kinematic parameters (N = 24). The variables “lower” and “upper” refer to the respective bounds of the 95% credible interval. [file peerj-06-6026-s001.docx]

| **Movement Time (ms)** | **M** | **SD** | **SE** | **Lower** | **Upper** |
| --- | --- | --- | --- | --- | --- |
| Left hand / compatible | 630 | 71 | 15 | 600 | 660 |
| Left hand / incompatible | 616 | 69 | 14 | 586 | 645 |
| Right hand / compatible | 584 | 65 | 13 | 557 | 612 |
| Right hand / incompatible | 642 | 72 | 15 | 611 | 672 |
|  |  |  |  |  |  |
| **Peak velocity (mm/s)** | **M** | **SD** | **SE** | **Lower** | **Upper** |
| Left hand / compatible | 1233 | 136 | 28 | 1176 | 1291 |
| Left hand / incompatible | 1289 | 169 | 34 | 1218 | 1360 |
| Right hand / compatible | 1325 | 157 | 32 | 1259 | 1392 |
| Right hand / incompatible | 1243 | 153 | 31 | 1178 | 1308 |
|  |  |  |  |  |  |
| **Time to peak velocity (ms)** | **M** | **SD** | **SE** | **Lower** | **Upper** |
| Left hand / compatible | 222 | 26 | 5 | 211 | 233 |
| Left hand / incompatible | 227 | 29 | 6 | 215 | 240 |
| Right hand / compatible | 223 | 24 | 5 | 213 | 234 |
| Right hand / incompatible | 225 | 26 | 5 | 214 | 236 |
|  |  |  |  |  |  |
| **Maximal grip aperture (mm)** | **M** | **SD** | **SE** | **Lower** | **Upper** |
| Left hand / compatible | 71 | 5 | 1 | 69 | 72 |
| Left hand / incompatible | 101 | 9 | 2 | 97 | 105 |
| Right hand / compatible | 102 | 8 | 2 | 99 | 106 |
| Right hand / incompatible | 71 | 7 | 1 | 68 | 74 |
|  |  |  |  |  |  |
| **Time to maximal grip aperture (ms)** | **M** | **SD** | **SE** | **Lower** | **Upper** |
| Left hand / compatible | 372 | 59 | 12 | 347 | 397 |
| Left hand / incompatible | 380 | 60 | 12 | 355 | 406 |
| Right hand / compatible | 361 | 55 | 11 | 338 | 385 |
| Right hand / incompatible | 395 | 62 | 13 | 368 | 421 |
