# Supplementary material for: Compatibility between object size and response side in grasping: the left hand prefers smaller objects, the right hand prefers larger objects
Source: PeerJ. 2018 Dec 3;6:e6026. doi: 10.7717/peerj.6026 (PMC6282946; doi:10.7717/peerj.6026)
Supplement: Table S2 — [P(M) indicates prior model probabilities which are set to be equal across all models. P(M—data) indicates the updated probabilities after having observed the data. BFM indicates the degree to which the data have changed the prior model odds. BF10 indicates the Bayes factor grading the intensity of the evidence that the data provide for H1 versus H0. Error % indicates the size of the error in the integration routine relative to the Bayes factor, similar to a coefficient of variation. All models include subject. [file peerj-06-6026-s002.docx]

| **Reaction time** |  |  |  |  |  |  |
| --- | --- | --- | --- | --- | --- | --- |
|  | **Models** | **P(M)** | **P(M\|data)** | **BF _M_** | **BF _10_** | **error %** |
|  | Null model (incl. subject) | 0.200 | 1.776e -5 | 7.105e -5 | 1.000 |  |
|  | Compatibility | 0.200 | 0.751 | 12.038 | 42257.258 | 1.592 |
|  | Response hand | 0.200 | 3.760e -6 | 1.504e -5 | 0.212 | 0.881 |
|  | Compatibility + Response hand | 0.200 | 0.159 | 0.756 | 8947.170 | 2.027 |
|  | Compatibility + Response hand + Compatibility  ✻  Response hand | 0.200 | 0.090 | 0.398 | 5092.980 | 1.914 |
|  | | | | | | |
| **Movement time** |  |  |  |  |  |  |
|  | Null model (incl. subject) | 0.200 | 5.104e -5 | 2.042e -4 | 1.000 |  |
|  | Compatibility | 0.200 | 2.212e -4 | 8.852e -4 | 4.334 | 1.226 |
|  | Response hand | 0.200 | 2.034e -5 | 8.138e -5 | 0.399 | 1.839 |
|  | Compatibility + Response hand | 0.200 | 9.314e -5 | 3.726e -4 | 1.825 | 2.784 |
|  | Compatibility + Response hand + Compatibility  ✻  Response hand | 0.200 | 1.000 | 10365.044 | 19583.156 | 2.902 |
|  | | | | | | |
| **Peak Velocity** |  |  |  |  |  |  |
|  | Null model (incl. subject) | 0.200 | 5.054e -4 | 0.002 | 1.000 |  |
|  | Compatibility | 0.200 | 1.559e -4 | 6.236e -4 | 0.308 | 5.729 |
|  | Response hand | 0.200 | 2.926e -4 | 0.001 | 0.579 | 1.290 |
|  | Compatibility + Response hand | 0.200 | 8.640e -5 | 3.456e -4 | 0.171 | 1.681 |
|  | Compatibility + Response hand + Compatibility  ✻  Response hand | 0.200 | 0.999 | 3841.048 | 1976.581 | 1.595 |
|  | | | | | | |
| **Time to peak velocity** |  |  |  |  |  |  |
|  | Null model (incl. subject) | 0.200 | 0.469 | 3.533 | 1.000 |  |
|  | Compatibility | 0.200 | 0.330 | 1.972 | 0.704 | 3.030 |
|  | Response hand | 0.200 | 0.100 | 0.442 | 0.212 | 1.030 |
|  | Compatibility + Response hand | 0.200 | 0.070 | 0.302 | 0.150 | 2.246 |
|  | Compatibility + Response hand + Compatibility  ✻  Response hand | 0.200 | 0.031 | 0.128 | 0.066 | 8.851 |
|  | | | | | | |
| **Maximal grip apeture** |  |  |  |  |  |  |
|  | Null model (incl. subject) | 0.200 | 2.326e -49 | 9.303e -49 | 1.000 |  |
|  | Compatibillity | 0.200 | 4.927e -50 | 1.971e -49 | 0.212 | 1.017 |
|  | Response hand | 0.200 | 5.092e -50 | 2.037e -49 | 0.219 | 1.434 |
|  | Compatibillity + Response hand | 0.200 | 1.061e -50 | 4.243e -50 | 0.046 | 1.951 |
|  | Compatibillity + Response hand + Compatibillity  ✻  Response hand | 0.200 | 1.000 | 1.165e +49 | 4.300e +48 | 3.253 |
|  | | | | | | |
| **Time to maximal grip aperture** |  |  |  |  |  |  |
|  | Null model (incl. subject) | 0.200 | 0.005 | 0.021 | 1.000 |  |
|  | Compatibility | 0.200 | 0.526 | 4.445 | 99.821 | 1.277 |
|  | Response hand | 0.200 | 0.001 | 0.005 | 0.217 | 1.104 |
|  | Compatibility + Response hand | 0.200 | 0.115 | 0.522 | 21.900 | 1.888 |
|  | Compatibility + Response hand + Compatibility  ✻  Response hand | 0.200 | 0.352 | 2.170 | 66.707 | 3.103 |
